# Phase Change Materials Meet Microfluidic Encapsulation

**DOI:** 10.1002/advs.202304580

**Published:** 2023-11-14

**Authors:** Yanhong Guo, Tuo Hou, Jing Wang, Yuying Yan, Weihua Li, Yong Ren, Sheng Yan

**Affiliations:** ^1^ Institute for Advanced Study Shenzhen University Shenzhen 518060 China; ^2^ Research Group for Fluids and Thermal Engineering University of Nottingham Ningbo China Ningbo Zhejiang 315104 China; ^3^ Department of Mechanical Materials and Manufacturing Engineering University of Nottingham Ningbo China Ningbo Zhejiang 315104 China; ^4^ Nottingham Ningbo China Beacons of Excellence Research and Innovation Institute University of Nottingham Ningbo China Ningbo Zhejiang 315104 China; ^5^ Department of Electrical and Electronic Engineering University of Nottingham Ningbo China Ningbo Zhejiang 315104 China; ^6^ Faculty of Engineering University of Nottingham Nottingham NG7 2RD UK; ^7^ School of Mechanical Materials Mechatronic and Biomedical Engineering University of Wollongong Wollongong 2522 Australia; ^8^ Key Laboratory of Carbonaceous Wastes Processing and Process Intensification Research of Zhejiang Province University of Nottingham Ningbo China Ningbo Zhejiang 315104 China; ^9^ College of Mechatronics and Control Engineering Shenzhen University Shenzhen 518060 China

**Keywords:** microencapsulated phase change capsules/fibers, microfluidics, phase change material, thermal energy storage

## Abstract

Improving the utilization of thermal energy is crucial in the world nowadays due to the high levels of energy consumption. One way to achieve this is to use phase change materials (PCMs) as thermal energy storage media, which can be used to regulate temperature or provide heating/cooling in various applications. However, PCMs have limitations like low thermal conductivity, leakage, and corrosion. To overcome these challenges, PCMs are encapsulated into microencapsulated phase change materials (MEPCMs) capsules/fibers. This encapsulation prevents PCMs from leakage and corrosion issues, and the microcapsules/fibers act as conduits for heat transfer, enabling efficient exchange between the PCM and its surroundings. Microfluidics‐based MEPCMs have attracted intensive attention over the past decade due to the exquisite control over flow conditions and size of microcapsules. This review paper aims to provide an overview of the state‐of‐art progress in microfluidics‐based encapsulation of PCMs. The principle and method of preparing MEPCM capsules/fibers using microfluidic technology are elaborated, followed by the analysis of their thermal and microstructure characteristics. Meanwhile, the applications of MEPCM in the fields of building energy conservation, textiles, military aviation, solar energy utilization, and bioengineering are summarized. Finally, the perspectives on MEPCM capsules/fibers are discussed.

## Graphical abstract

This review provides an overview of microfluidic microencapsulation of phase change materials (PCMs), the applications of microencapsulated PCMs in the fields of building energy conservation, textiles, military aviation, solar energy utilization, and bioengineering, and the future perspectives on microencapsulated PCMs capsules/fibers.

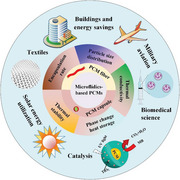

## 1 Introduction

As the economy grows rapidly, there is a rising global demand for energy. However, the continuous development of nonrenewable energy has had an irreversible impact on the environment, and the problem of energy shortage has become increasingly serious. Therefore, in recent years, the issue of how to effectively improve the energy utilization rate has aroused widespread concern, among which new energy storage materials are the current research focus. Phase change materials (PCMs) exhibit exceptional heat storage capabilities, efficiently absorbing and releasing substantial energy during phase transitions.^[^ ^1^ ^]^ This renders them invaluable across an array of applications, including optimizing photovoltaic system heat control,^[^ ^2^ ^]^ enhancing energy storage in supercapacitors,^[^ ^3^ ^]^ batteries,^[^ ^4^ ^]^ and electronic devices.^[^ ^5^ ^]^ PCMs also offer potential in regulating temperatures in buildings^[^ ^6^ ^]^ and medical sectors.^[^ ^7^ ^]^ Moreover, they empower the creation of thermoregulating fabrics^[^ ^8^ ^]^ and the effective utilization of residual and renewable thermal energy in diverse processes such as drying, water desalination, and heating.^[^ ^9^ ^]^

Although PCMs have been widely used as a heat storage medium, in practical applications, pure PCMs have defects as they are vulnerable to leakage, corrosion, phase separation, volume change, etc.^[^ ^10^ ^]^ In contrast, if microencapsulation/microfiber packaging is carried out before PCMs are used for energy storage, due to the encapsulation of solid shells, microencapsulated phase change materials (MEPCM) capsules/fibers have greater advantages over unpackaged capsules/fibers in terms of storage, transportation, and application. Besides, as a solid shell can prevent the interaction of nuclear PCMs with the outside environment, it can not only improve the stability, specific surface area, and heat transfer efficiency of the unpacked PCM, but also solve the problems of PCM leakage, phase separation, and corrosivity.

In fact, because of the above advantages of MEPCM capsules/fibers, they have currently been widely used in the fields of building energy conservation,^[^ ^11^ ^]^ thermal storage and temperature regulation textiles,^[^ ^12^ ^]^ military,^[^ ^13^ ^]^ and functional thermal fluids.^[^ ^14^ ^]^ Until now, many methods have been developed to fabricate MEPCM capsules/fibers. **Table** **1** shows the conventional microencapsulation methods of PCMs. On the one hand, the early physical methods caused violent flows, poor microcapsule uniformity, and limitations in core–shell material selection,^[^ ^15^ ^]^ and chemical methods restrict material choices and require highly reactive monomers. On the other hand, the traditional MEPCM fiber preparation lacks precise control, resulting in incomplete core–shell structures, PCM leakage, and reduced phase‐change latent heat.^[^ ^16^ ^]^

**Table 1:** Conventional microencapsulation methods of PCMs.

Physical synthesis methods	Physical chemical synthesis methods	Chemical synthesis methods
Electrostatic encapsulation^[^ ^17^ ^]^	Sol–gel encapsulation^[^ ^15a^ ^]^	Suspension polymerization^[^ ^18^ ^]^
Spray‐drying^[^ ^19^ ^]^	Coacervation^[^ ^15b^ ^]^	In situ polymerization^[^ ^20^ ^]^
One‐step method^[^ ^21^ ^]^	Supercritical CO_2_‐assisted^[^ ^22^ ^]^	Emulsion polymerization^[^ ^23^ ^]^
–	–	Dispersion polymerization^[^ ^24^ ^]^
–	–	Interfacial polymerization^[^ ^25^ ^]^

**Table 2:** Advantages, disadvantages, and applications of PCMs.^[^ ^34^, ^38^ ^]^

Types of materials	Advantages	Disadvantages	Applications
Organics PCMs	Large temperature range	Low thermal conductivity	Thermal energy storage
	Compatible with other materials	Flammable	Building and construction industry
	No supercooling	Relatives large volume changes	Electronics thermal managements
	No separation	Expensive except technical grade paraffin wax	Cold storage and transportation
	Chemically stable		Textile industry
	Safe		Electronics thermal managements
	Nonreactive		Automotive industry
	Easily recycled		Aerospace industry
			Energy‐efficient refrigeration
			cosmetics
			Food industry
			Biomedical fields
Inorganic PCMs	High volumetric latent heat	High changed volume	Thermal energy storage
	Easily available	Supercooling	Building and construction industry
	Less expensive	Corrosiveness	Electronics thermal managements
	Higher thermal conductivity		Cold storage and transportation
	High thermal fusion		Textile industry
	Lower volumetric variation		Electronics thermal managements
	Nonflammable		Automotive industry
			Aerospace industry
			Energy‐efficient refrigeration
Eutectics	Sharp melting point	Limited thermophysical properties data	Thermal energy storage
	High volumetric storage density		Building and construction industry
			Cold storage and transportation
			Electronics thermal managements
			Energy‐efficient refrigeration

In recent years, microfluidic technology is considered to be a favorable tool for preparing functional material precursors by virtue of its ability to accurately control a small amount of fluids in microchannels with small cross‐sectional dimensions.^[^ ^26^ ^]^ It can be seen from previous studies^[^ ^27^ ^]^ that microfluidic technology can accurately control the formation and stability of fluid interface. On the basis of obtaining multiple emulsion templates and laminar liquid lines, precise and controllable microcapsules and microfiber structures can be obtained by reacting or curing at the phase interface, which can be helpful to confine corrosive or toxic materials, enclose powder materials, and control the release of different compounds. Due to these advantages, microencapsulation has been employed for the confinement of a wide range of compounds that include: bacteria, drugs, and cells in the pharmaceutical and biomedicinal field; additives used in cosmetic and food industries.^[^ ^28^ ^]^ Specially, with the unique advantages of microfluidic technology in the field of controllable preparation of microcapsules and microfiber functional materials, through precise control of the structure of MEPCM capsules/fibers, the encapsulation rate and monodispersity of MEPCM capsules/fibers can be effectively improved.

**Figure 1:**
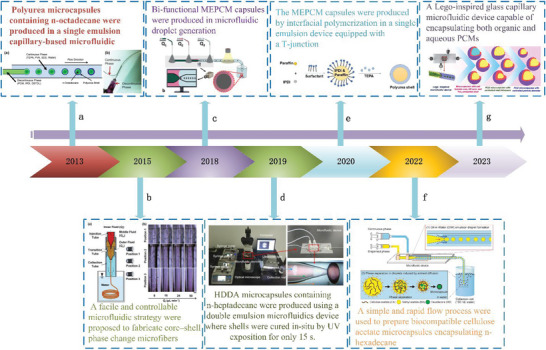
Timeline of the development history for MEPCM capsules/fibers. a) Reproduced with permission.^[^ ^57^ ^]^ Copyright 2013, Elseiver. b) Reproduced with permission.^[^ ^77^ ^]^ Copyright 2015, Elsevier. c) Reproduced with permission.^[^ ^100^ ^]^ Copyright 2018, American Chemical Society. d) Reproduced with permission.^[^ ^61^ ^]^ Copyright 2019, Elseiver. e) Reproduced with permission.^[^ ^70^ ^]^ Copyright 2020, Wiley. f) Reproduced with permission.^[^ ^73^ ^]^ Copyright 2022, Elsevier. g) Reproduced with permission.^[^ ^72^ ^]^ Copyright 2023, Elsevier.

Many reviews have examined the fabrication of MEPCM capsules/fibers.^[^ ^1^, ^29^ ^]^ Salaün^[^ ^30^ ^]^ highlighted the coacervation, interfacial, and in situ polymerization techniques, exploring advancements to bolster thermal characteristics of MEPCM capsules. Zhao and Zhang^[^ ^29^ ^]^ covered interfacial, suspension, and in situ polymerization methods, including complex coacervation. They also discussed MEPCMs' use in textiles and construction. However, these previous assessments predominantly concentrated on conventional PCM encapsulation approaches, leaving room for exploration of newer methodologies. Few article reviewed the methods and characteristics of MEPCM capsules/fibers based on microfluidic device. Gao et al.^[^ ^31^ ^]^ provided insights on microfluidics‐based MEPCM capsules/fibers, but primarily focuses on the limited applications in the fields of thermal regulation, thermal response, and wearable electronics. Therefore, it is necessary to provide a comprehensive review on the recent progress on the fabrication, properties, and applications of microfluidics‐based capsules/fibers. Significantly, there were some landmark nodes in the development history of microfluidics‐based capsules/fibers, as illustrated in **Figure** **1**.

**Figure 2:**
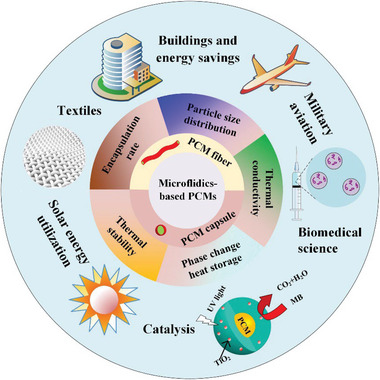
The outline of microfluidics‐based PCMs from fabrication to applications.

On this basis, we first introduced the geometry of microfluidic devices that can be used to prepare MEPCM capsules/fibers. In addition, the theoretical model of MEPCM capsules/fibers is discussed, and the general preparation and properties of MEPCM capsules/fibers reported so far are summarized. Finally, we emphasized the characterization of microfluidics‐based MEPCM capsules/fibers and their applications, including the fields of building, textiles, solar energy utilization, military aviation, catalysis, and bioengineering, and finally provided insights on the future direction of this field (**Figure** **2**).

## 2 Classification of PCMs

**Figure 3:**
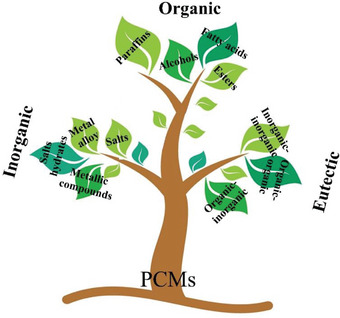
Classification of PCMs.

PCMs can be categorized into three primary types based on their chemical composition: inorganic, organic, and eutectic PCMs,^[^ ^32^ ^]^ as illustrated in **Figure** **3**.

Within the organic PCM category, a further subdivision can be made into two sub‐categories: paraffin and nonparaffin compounds.^[^ ^32^ ^]^ Paraffin PCMs are composed of linear alkanes, characterized by the molecular formula C*_n_* H_2_ *_n_*_+2_. These materials offer several key advantages, including substantial heats of fusion, chemical stability, safety, compatibility with noncorrosive metal containers, and cost‐effectiveness.^[^ ^33^ ^]^ Their versatility allows for compatibility with a wide range of encapsulating materials, making them a popular choice.^[^ ^34^ ^]^ Nonparaffin PCMs represent the largest group and encompass fatty acids, fatty alcohols, and esters. Fatty acids are typically carboxylic acids consisting of long aliphatic chains, which can be saturated or unsaturated. Fatty alcohols, on the other hand, are primarily linear‐chain primary alcohols with carbon atoms ranging from 4–6 to 22–26.^[^ ^35^ ^]^ Despite their distinct characteristics, nonparaffin PCMs share common features, including a high heat of fusion, the ability to undergo phase changes without supercooling, widespread availability, and cost‐effectiveness.^[^ ^33^ ^]^ These qualities render them suitable for numerous applications.^[^ ^36^ ^]^

Inorganic PCMs, in contrast to their organic counterparts consisting of carbon, hydrogen, and oxygen atoms, are composed of non‐carbon‐based substances such as salt hydrates, salts, metallic compounds, and metal alloys. While these materials offer higher energy storage density and thermal conductivity compared to organic PCMs, they do come with certain drawbacks, including issues with corrosion and a propensity for supercooling. Additionally, their operational temperature range is generally higher when compared to organic PCM options.^[^ ^36^, ^37^ ^]^

Eutectic PCMs refer to mixtures comprising two or more compounds, typically a combination of organic–organic, organic–inorganic, or inorganic–inorganic materials. A notable advantage of eutectic PCMs lies in their ability to attain specific melting points by adjusting the proportions of each component within the mixture. In a eutectic PCM, the blending of two or more materials results in a substance with a lower melting point than any individual component. Upon heating, this material melts and absorbs heat, and upon cooling, it solidifies and releases heat. The eutectic composition provides a well‐defined melting point, enhancing the PCM's efficiency in storing and releasing thermal energy.^[^ ^34^ ^]^ A material's viability as a PCM in various applications hinges on its apt phase change temperature. However, thermal, physical, kinetic, chemical, and economic attributes also significantly impact PCM performance, storage system traits, and application feasibility. **Table** **2** encompasses crucial properties and the applications for PCMs.

## 3 Microfluidic‐Based Double Emulsion/Liquid Line Template Formation

The double‐phase microfluidic encapsulation method is developed based on microfluidic technology, which has the advantage of precise manipulation of low flow fluid interfaces in microchannels. It is considered as a favorable tool for the preparation of functional materials, such as those applied in the fields of pharmaceuticals, foods, and phase change energy storages.^[^ ^27^, ^39^ ^]^ The key to the preparation of functional materials by double‐phase microfluidic encapsulation is to first form double emulsion and laminar liquid lines with highly controllable structure, high coverage, and good monodispersity through microfluidic technology.^[^ ^27a^ ^]^

### 3.1 Double Emulsion Formation

**Figure 4:**
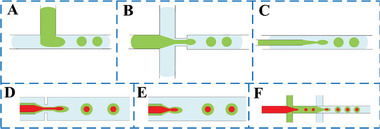
Junction geometries, A) cross‐flow, B) flow‐focusing, and C) co‐flow. D) Flow‐focusing microfluidic device.^[^ ^42^ ^]^ E) Coaxial co‐flow microfluidic device.^[^ ^43^ ^]^ F) Dual cross‐sectional microfluidic device.^[^ ^44^ ^]^

Microfluidic technology is characterized by small size, automation, and high integration. It enables the stable and continuous preparation of monodisperse droplets even under low flow rate conditions. It can precisely control the size and structure of a single droplet to ensure the high encapsulation rate of internal droplets. Therefore, microfluidic technology shows incomparable advantages in the continuous and controllable generation of double emulsion. On the one hand, microfluidic chips can be categorized into 2D and 3D based on their structure and design, which have been developed for preparing double emulsion based on their applications. 2D microfluidic chips are usually fabricated in silicon and glass substrates using lithography and etching techniques, or in polymer substrates such as polydimethylsiloxane (PDMS) by soft lithography,^[^ ^40^ ^]^ while 3D microfluidic chips are usually assembled and constructed by glass capillaries. On the other hand, microfluidic chips are classified into noncoaxial and coaxial based on their functionality and the arrangement of fluid flow within the chip. The noncoaxial T‐shaped cross‐flow microfluidic device^[^ ^41^ ^]^ is shown in **Figure** **4**A, where the flow directions of the internal and external fluids are perpendicular to each other. The internal fluid breaks into droplets at the intersection of the T‐shaped flow channel mainly due to the shearing and extrusion caused by the external fluid. Figure 4B,C shows two typical coaxial flow forms: co‐flow type and flow‐focusing type. In this device, the flow directions of internal phase and external phase fluids are located on the same axis, and the fracture generation of droplets in this device is generally affected by the viscous and shear stress of internal phase and external phase fluids.

Until now, researchers have studied the stable formation of double and multiple emulsions in microfluidic devices. Utada et al.^[^ ^42^ ^]^ constructed a flow‐focused microfluidic device, as shown in Figure 4D. The experiment showed that multiemulsion prepared by the device has high monodispersity and the multiemulsion with different shell thickness and different number of inner core droplets can be controlled by adjusting the three‐phase flow rate independently. In order to obtain double emulsion with an ultra‐thin shell, Kim et al.^[^ ^45^ ^]^ reformed the above device and placed the internal phase tube inside the mesophase tube, so that the internal phase can form a jet in the mesophase during the flow process and then pass through the focusing hole with the external phase, thus realizing the high‐frequency preparation of double emulsion with a thickness of only tens of nanometers. In addition to the flow‐focusing device, the 3D coaxial co‐flow microfluidic device is also widely used in emulsion preparation. Fischer et al. and Utada et al.^[^ ^46^ ^]^ studied the influence of physical parameters and velocity of each phase on droplet generation in coaxial microfluidic devices through experiments, and observed two typical flow patterns and their conversion laws. On the basis of elucidating the droplet formation mechanism and the evolution law of main flow patterns in the co‐flow device, various co‐flow microfluidic devices for generating multiple emulsions with complex structures are constantly emerging. Chu et al.^[^ ^43^ ^]^ assembled a 3D co‐flow microfluidic device in multiple stages, as shown in Figure 4E. First, they obtained double emulsion with highly controllable internal droplet nuclei. On this basis, adding a first‐stage co‐flow device can realize the regulation of the droplet size and the number of nuclei in the triple emulsion, and at the same time, it can ensure that the coefficient of variation (CV) value of the prepared multiple emulsion is less than 1.5%. Wu et al.^[^ ^47^ ^]^ compared the differences between the two experimental devices of co‐flow and flow‐focusing in the production of the simplest dual lotion through experiments. The results showed that the co‐flow microfluidic device has the advantages of a stable production process and good repeatability in the preparation of double emulsion. More importantly, its monodispersity in the production of double emulsion is better than that of the flow‐focusing microfluidic device. In addition to the flow‐focusing and co‐flow microfluidic device, the cross‐sectional microfluidic device has also been used for producing double emulsions. Pannacci et al.^[^ ^44^ ^]^ found through experiments that the cross‐channel has better control over the generation of double emulsion droplets than the T‐channel, as shown in Figure 4F. Saeki et al.^[^ ^48^ ^]^ found that if both cross‐channels have hydrophobic walls, and the depth of the first channel is the same as that of the central main channel, but smaller than the depth of the second channel, ultra‐thin (<1 µm) W/O droplets can be formed.

In addition to the experimental study, the hydrodynamic behavior of double and multiemulsion formation in microfluidic devices has also been numerically simulated to provide insights into the droplet production mechanism.^[^ ^49^ ^]^ Zhou et al.^[^ ^50^ ^]^ combined the finite element method with an adaptive grid to carry out numerical simulation and studied the formation process of single droplet and double emulsion in a flow‐focusing microfluidic device and their corresponding influencing factors. It was found that the encapsulation state and encapsulation efficiency of composite droplets in the device depended on the viscosity ratio and capillary number. Vu et al.^[^ ^51^ ^]^ used a forward tracking/finite difference method to track the unsteady evolution and rupture process of composite jet under Navier–Stokes program control system of incompressible Newtonian fluid. The results showed that the Reynolds number and Weber number of the internal phase fluid determined whether the composite jet breaks down in a drop mode or a spray mode to form droplets. In addition, the influence of velocity ratio (mesophase velocity ratio internal phase velocity, external phase velocity ratio internal phase velocity) on flow pattern transformation is also discussed. Liu et al.^[^ ^47^ ^]^ compared the formation mechanism and process of multiple emulsions in two axisymmetric coaxial microfluidic devices by numerical simulation and expounded the influence of flow conditions and local geometry of the device on the formation of double emulsions. It was found that the existence of focusing holes in the flow‐focusing microfluidic device was helpful to accelerate the fracture of the neck to form double emulsions under drip flow pattern and promote the formation of jet segments under spray flow pattern. The radius of focusing hole had an important influence on the formation of double emulsion, but the length of focusing hole had little influence on it. At the same time, the relationship between the dimensionless focusing hole radius and the capillary number of continuous phase fluid and the generated flow pattern in microfluidic devices was quantitatively described. Based on the above analysis, it can be seen that there have been many experiments and numerical simulation studies on the stable generation of multiple emulsion templates in microfluidic devices and their precise structural regulation, which provide important guidance for the related research on controllable preparation of microspheres and microcapsule materials with good structural size uniformity.

### 3.2 Laminar Liquid Line Template Formation

**Figure 5:**
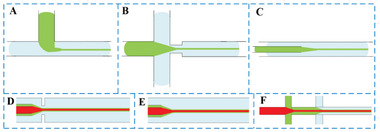
Laminar liquid line template formation. Junction geometries, A) cross‐flow, B) flow‐focusing, and C) co‐flow. D) Flow‐focusing microfluidic device.^[^ ^55^ ^]^ E) Coaxial co‐flow microfluidic device. F) Dual cross‐sectional microfluidic device.

In addition to producing double emulsion droplets, microfluidic technology has been considered as a favorable tool for continuous preparation of laminar liquid lines of functional microfiber materials by virtue of precise manipulation of fluids in noncoaxial T‐shaped cross‐flow, coaxial co‐flow, and coaxial flow‐focusing microfluidic device, as shown in **Figure** **5**A–C. Guillot et al.^[^ ^52^ ^]^ used the theory of absolute instability and convection instability to explain the stability of laminar liquid line formation in the cross‐flow process. In simple terms, absolute instability means that the disturbance will grow and spread from a fixed point to the upstream and downstream directions. In this case, the jet cannot be formed continuously but breaks into droplets. On the contrary, the interference in the so‐called convective instability only grows and propagates in the downstream direction, which allows a long continuous liquid line to persist. Research has shown that this situation generally occurs when the working fluid velocity is large and the inertia effect of the fluid is more important than the surface tension effect.^[^ ^52^ ^]^ The preparation process of double emulsion templates in microfluidic chips is related to many parameters such as flow velocity, viscosity, interfacial tension, and device geometry. Humphry et al.^[^ ^53^ ^]^ found that when the width of the dispersed phase jet section is equal to or greater than the channel height in the device, its instability can be suppressed. The geometric constraints of the device can be used to suppress jet instability, promoting the stable generation of laminar liquid lines. Additionally, experimental research^[^ ^54^ ^]^ showed that the tensile viscosity of non‐Newtonian fluid as a dispersed phase solution resists the extrusion required to form droplets, leading to the formation of long jet state. The dispersed phase of non‐Newtonian fluid with high viscosity could effectively suppress the instability caused by the generation of laminar liquid lines in the flow‐focusing device. Recently, in order to obtain fiber materials with structural diversity, higher requirements have been put forward for the construction of laminar liquid lines in the flow‐focusing, co‐flow, or flow‐focusing microfluidic device, as shown in Figure 5D–F. Yu et al.^[^ ^55^ ^]^ used a glass capillary to construct various microfluidic devices with special structures to form multipattern laminar liquid lines, as shown in Figure 5D, making it possible to prepare microfibers in different fields. This review mentions that multiple capillaries can be used in parallel as internal injection tubes, allowing various fluids flowing in the capillaries to not mix with each other before entering the collection channel for solidification, so microfibers with multichamber structure can be prepared. A laminar liquid line with nested structure can be generated by nesting and assembling multiple capillaries as internal injection tubes. At present, theoretical and experimental research on the factors affecting the stability of laminar liquidus has been quite rich, and there have been related studies on the construction of laminar liquidus with different structures. These flexible flow forms can be applied to the creation of ultrafine fibers with novel 3D structures.

### 3.3 Heat Transfer Model of MEPCMs

**Figure 6:**
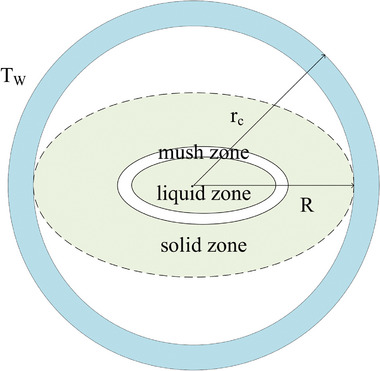
Simplified model of PCM capsule.

**Figure** **6** shows the physical model of the PCM capsule, which comprises a sphere PCM core encapsulated by shell material. When the phase change of PCM core occurs within a certain temperature range, there will be three zones with different states, including a solid zone, mushy zone, and liquid zone, within the material. In dealing with the melting process of a multicomponent core‐and‐shell MEPCM capsule, a mathematical model is constructed based on specific assumptions.^[^ ^31^, ^56^ ^]^ 1) The core encompasses both PCM and doping elements, 2) with the capsule's size enabling the omission of thermal convection effects in the PCM's liquid phase; 3) the core PCM experiences solid and liquid states with consistent thermophysical traits, alongside a mushy zone characterized by evolving properties. Notably, 4) the shell's characteristics remain unaffected by PCM volume changes. 5) Homogeneity characterizes the capsule's inner region.

Leveraging these assumptions, the moving boundary problem is addressed using the apparent heat capacity method.^[^ ^31^, ^56^ ^]^ The ensuing model encapsulates the entire MEPCM capsule, with the energy equation expressed in spherical coordinates as

(1) $$C^{*}\;\frac{\partial T}{\partial t}=\frac{1}{r^{2}}\;\frac{\partial }{\partial r}\left(r^{2}\lambda^{*}\frac{\partial T}{\partial r}\right)$$

where C* is the effective thermal capacity (J kg^−1^ K^−1^) of the MEPCM capsule, *T* is the temperature (K), *t* is the time (s), *r* is the radius (m), and λ* is the effective thermal conductivity (W m^−1^ K^−1^). With an initial temperature of *T*_0_, the MEPCM capsule's outer shell temperature is elevated to *T*_s_ due to an external heat source. The boundary condition can be expressed as

(2) $$T{|}_{t=0}=T_{0},\;0\leq r\leq r_{c}$$

(3) $$\frac{\partial T}{\partial r}{|}_{r=0}=0,\;t>0$$

(4) $$T{|}_{r=r_{c}}=T_{s},\;t>0$$

where *r*_c_ is the radius of the core. The effective thermal capacity C* in Equation (1) can be given as

(5) $$C^{*}=\left\{\begin{array}{cc} \sum m_{c}C_{c}, & 0\leq r\leq r_{c}\\ C_{s}, & r_{c}\leq r\leq r_{\mathrm{cap}} \end{array}\right.$$

where *m*_c_ and *C*_c_ denote the mass and thermal capacity of individual core components, while *C*_s_ represents the thermal capacity of the shell, and *r*_cap_ stands for the radius of the MEPCM capsule. Within the core's components, the effective thermal capacity of the PCM (*C*_p_) necessitates consideration of latent heat across the phase change temperature range (*T*_p_ + Δ*T*). This can be expressed as follows

(6) $$C_{p}=\left\{\begin{array}{cc} C_{p,s}, & T<\left(T_{p}-\Delta T\right)\\ \frac{\rho_{p}H_{p}}{2\Delta T}+\frac{C_{p,s}+C_{p,l}}{2}, & \left(T_{p}-\Delta T\right)\leq T\leq \left(T_{p}+\Delta T\right)\\ C_{p,l}, & T>\left(T_{p}-\Delta T\right) \end{array}\right.$$

here *C*_p, s_ and *C*_p, l_ represent the thermal capacity of the PCM in its solid and liquid states, respectively. ρ_p_ pertains to the effective density of the PCM for both phases, while *H*_p_ signifies the PCM's latent heat. *T*_p_ stands for the average phase change temperature, and Δ*T* corresponds to half of the phase change temperature difference. Moreover, the expression for effective thermal conductivity, λ*, as in Equation (1), can be formulated as follows

(7) $$\lambda^{*}=\left\{\begin{array}{cc} \lambda_{c}, & 0<r\leq r_{c}\\ \lambda_{s}, & r_{c}<r\leq r_{\mathrm{cap}} \end{array}\right.$$

here λ_c_ and λ_s_ represent the effective thermal conductivity of the core and shell, respectively. In the case of MEPCM fibers, the energy equation and associated boundary conditions are transformed into a framework based on cylindrical coordinates.

## 4 Fabrication of MEPCMs Capsules/Fibers

From the above research, it can be seen that microfluidic technology can accurately control the formation and stability of fluid interface. MEPCM capsules/fibers with precisely controllable microstructure can be obtained by reacting or curing at the external interface of double emulsion and laminar liquidus, based on the two types of microfluidic encapsulated templates with stable phase interface.

### 4.1 Fabrication of MEPCM Capsules

Researchers have done a lot of experimental and simulation studies on the formation of double emulsion in microfluidic devices. At present, the microfluidic encapsulation method based on double emulsion has become an important method for preparing MEPCM capsules.

**Figure 7:**
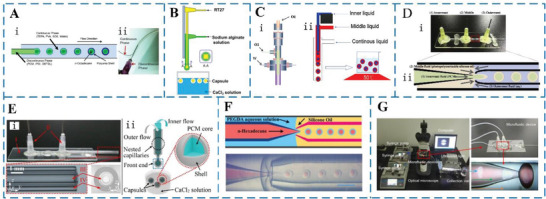
A) i) Schematic diagram of the process of creating monodisperse PCM@polyurea microcapsules in a tubular microfluidic device and ii) photograph of the oil‐in‐water PCM droplets produced at the tubular junction. Emulsification and partial polycondensation of the PCM and polyurea in the flow stream at 35 °C. Reproduced with permission.^[^ ^57^ ^]^ Copyright 2013, Elsevier. B) Schematic illustration of the equipment and process used to prepare RT27/Ca‐alginate capsules. Reproduced with permission.^[^ ^58^ ^]^ Copyright 2014, Elsevier. C) i) The microfluidic device and ii) the process used to fabricate double emulsions. Reproduced with permission.^[^ ^59^ ^]^ Copyright 2014, Elsevier. D) i) The glass capillary device and ii) the process used to prepare double emulsion droplets as templates for the MEPCM capsules. Reproduced with permission.^[^ ^60^ ^]^ Copyright 2019, Elsevier. E) Gravity‐assisted co‐flowing microfluidic device (I: glass slide, II: dispensing needles, III: inner capillary, IV: outer capillary). Reproduced with permission.^[^ ^63^ ^]^ Copyright 2022, Elsevier. F) Schematic of microfluidics device. Reproduced with permission.^[^ ^62^ ^]^ Copyright 2020, American Chemical Society. G) Three‐phase microfluidic device for the fabrication of MEPCM capsules. Reproduced with permission.^[^ ^61^ ^]^ Copyright 2019, Elsevier.

Based on a tubular microfluidic technology, Lone et al.^[^ ^57^ ^]^ prepared an MEPCM capsules with a core material of n‐octadecane and a shell material of polyuria, as shown in **Figure** **7**A. The results showed that the monodisperse particle size of MEPCM capsules could be controlled by changing the flow rate or aqueous solution. However, the polyurea shell is too thin and wrinkled, making it difficult to provide effective protection for the MEPCM capsules under thermal cycling conditions, possibly because the strict time and temperature requirements of the thermal curing process cannot be met in microfluidic devices. Therefore, new microfluidic technologies were further developed to manufacture double emulsion templates for generating MEPCM capsules.^[^ ^64^ ^]^ For example, Liang et al.^[^ ^58^ ^]^ developed a simple and easy‐to‐control method for encapsulating PCM with calcium alginate, a biocompatible and nontoxic material. This was achieved using a microfluidic device, as shown in Figure 7B. The study found that adjusting the inner flow rate was more effective than adjusting the outer flow rate for controlling the size of the MEPCM capsules and the content of RT27, which is a type of PCM. Besides, the MEPCM capsules produced using this method showed good thermoregulating ability and were stable and reproducible. Similarly, Fu et al.^[^ ^59^ ^]^ prepared MEPCM capsules with silicone resin as the shell and n‐cetyl bromide as core based on co‐flow microfluidics, as shown in Figure 7C. The findings of the study indicated that the microcapsules had a high energy storage capacity. Specifically, the melting enthalpy and freezing enthalpy of the microcapsules were measured to be 76.35 and 78.67 J g^−1^, respectively. Moreover, the thermal curing process used in the study mentioned earlier^[^ ^58^ ^]^ typically requires several hours to complete the polymerization process.^[^ ^65^ ^]^ However, an alternative method for polymerization is UV radiation curing, which is a photopolymerization technology that enables liquid resins to be rapidly converted into solid polymers without the use of solvents. Many studies have shown that UV curable materials have better mechanical properties than thermal curing materials.^[^ ^66^ ^]^ Based on this, Akamatsu et al.^[^ ^60^ ^]^ used a simple capillary microfluidic method (Figure 7D) to prepare encapsulated n‐tetradecane and n‐hexadecane in a silicone resin shell with the help of UV curing. The study demonstrated that the MEPCM capsules produced were suitable for thermal energy storage, as the stable phase transition of the capsules was directly observed. In a similar study, Li et al.^[^ ^61^ ^]^ used a co‐flow capillary microfluidic device and UV curing technique (as shown in Figure 7E) to prepare MEPCM capsules with n‐heptane as the core and hexanediol diacrylate (HDDA) polymer as the shell. The resulting MEPCM capsules had a uniform particle size, smooth surface, and regular spherical shape. In addition, in order to explore the possibility of core materials for MEPCM capsules, Han et al.^[^ ^62^ ^]^ proposed a microfluidic device (Figure 7F) for the precise manufacture of MEPCM capsules with adjustable thermal properties. The study found that both organic and inorganic MEPCM capsules had excellent properties such as high monodispersity, good energy storage capacity, high encapsulation efficiency, and thermal stability. Consequently, these capsules are reliable for thermal energy storage applications. It should be noted that the PCM core material used in the above studies^[^ ^57^, ^58^, ^59^, ^60^, ^62^ ^]^ were pure materials without any additives.

**Figure 8:**
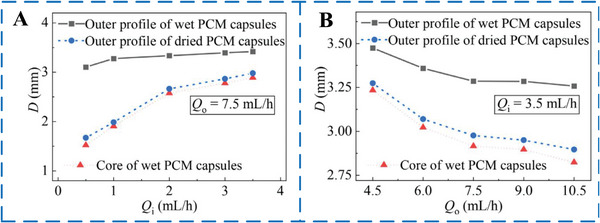
The relationship between the detailed diameters on the rates of the A) inner flow *Q*_i_ and B) outer flow *Q*_o_. Reproduced with permission.^[^ ^63^ ^]^ Copyright 2022, Elsevier.

However, the low thermal conductivity of commonly used PCM inhibits the overall thermal performance, such as thermal storage, thermal response, and heat release speed of MEPCM capsules in practical applications.^[^ ^29^ ^]^ Therefore, in order to improve the heat conductivity of MEPCM capsules, Hao et al.^[^ ^63^ ^]^ prepared microcapsules based on the microfluidic method (Figure 7G). To enhance the thermal conductivity of the pure PCM and improve the thermal regulation ability of the MEPCM capsules, multilayer graphene was added to the PCM. The study found that the resulting microcapsules had a stable core–shell structure and high monodispersity. The size and core/shell ratio, which determines the thermal adjustment capability, could be accurately controlled by adjusting the flow rates of the inner and outer phases (as shown in **Figure** **8**A,B). Additionally, the addition of multilayer graphene (up to 2 wt%) improved the thermal conductivity of the microcapsules, while the energy storage capacity degradation was less than 5%. This allowed the MEPCM capsules to respond faster to changes in the surrounding thermal environment. Although all of these microfluidic devices can produce uniform and high packaging rate phase change PCMs, they are difficult to manufacture. In common glass capillary devices, not only is it troublesome, but also due to the adhesion between the capillary and the needle, it is impossible to adjust the internal structure during the preparation process.^[^ ^67^ ^]^ In addition, PDMS channels not only rely heavily on expensive silicon master modules, but also are sensitive to organic solvents.^[^ ^68^ ^]^ To solve these problems, Parvate et al.^[^ ^72^ ^]^ proposed a Lego‐inspired microfluidic device to produce MEPCM capsules with a movable UV polymerization. The microfluidic device used in the study consisted of a coaxial glass capillary and computer numerical control‐milled blocks that could be easily connected and taken apart using a stud‐and‐tube system inspired by Lego blocks. The study found that the thickness of the shell and the diameter of the particles in the microcapsules could be accurately controlled by adjusting the fluid rate and capillary geometry. **Table** **3** provides a summary of the morphological characteristics and thermal properties of some MEPCM capsules that were prepared using microfluidic methods.

**Table 3:** Thermal properties and morphological characteristics of MEPCM capsules.

Ref.	Microfluidic	Device	Emulsions	Phases	Curing method	Core material	Shell material	Capsule size [µm]	Δ*H*_m_ [J g^−1^]	*T*_m_ [°C]	Δ*H*_c_ [J g^−1^]	*T*_c_ [°C]	*W* [%]	*E* [%]	CV
[60]	Flow‐focusing	Glass capillary	Double	O/O/W	UV radiation	n‐tetradecane/n‐hexadecane	Photopolymerizable oil	120–200	96	19.1		8.4	44		High monodispersity
[69]	Flow‐focusing	Glass capillary	Double	W/O/W	UV radiation	sodium acetate trihydrate aqueous solution	Acrylate/polystyrene	250–280	–	1.7	–	−48.3	–		Poor concentricity
[62]	Flow‐focusing	Glass capillary	Double	O/WO and W/O/O	UV radiation	n‐hexadecane	PEGDA/ETPTA	200–300	269.3 J g^−1^	19.1/8	212.5	5.8/−24	87.8	82.9	<2%
[70]	T‐junctions	Tubular	Single	O/W	Interfacial polymerization	Paraffin	Polystyrene	500	87.5	29.1	94.9	24.5	96.5	96.0	<2.5%
[59]	Co‐flow + collector	Tubular	Double	O/O/W	Chemically crosslinked	n‐hexadecyl bromide	Elastic silicone	460	76.35	16	78.67	6	49	–	<3%
[71]	Co‐flow	Glass capillary	Single	W/O	Sol–gel	Na_2_SO_4_⋅10H_2_O	SiO_2_ microparticles	400–600	–	34.71		25.89	–	47.07%	2.79%
[57]	Co‐flow	Glass capillary	Single	O/W	In situ polycondensation	n‐octadecane	Polyurea	35–500	169.7	35	165.7	25	–	–	<3%
[58]	Co‐flow	Glass capillary	Single	O/W	Physically crosslinked	RT27	Ca‐alginate	2600–4200	179.4	27	178.5	27	–	94.68%	<3%
[61]	Co‐flow	Glass capillary	Double	O/O/W	UV radiation	n‐heptadecane	HDDA	30–150	161.6	8 and 21.2	159.7	7.6 and 21.3	73.72	73.36	<2%
[63]	Co‐flow	Glass capillary	Single	W/O	Calcium chloride solution (CaCl_2_)	Multilayer graphene and RT25	Calcium chloride	1500–3500	150.8	26	153.5	22	84.2		<2%
[72]	Co‐flow	Glass capillary	Double	O/O/W and W/O/W	UV radiation	Hexadecane or salt hydrate SP21EK	Norland optical adhesive	250–260	157	20.3	158	16.4	65.4	62.0	<3%
[73]	Co‐flow	Glass capillary	Single	O/W	Crosslinking	n‐hexadecane	Cellulose acetate	45–89	178	16.9	176	18.5	66		<11.3%
[74]	Co‐flow	Glass capillary	Double	W/O/W	UV radiation	OP18E	HDDA	about 475	19.8	19.09	18.12	12.18			

### 4.2 Fabrication of MEPCM Fibers

**Figure 9:**
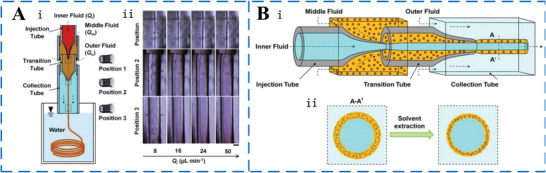
A) The setup of coaxial microfluidic device and generation process of MEPCM fibers. i) Schematic diagram of microfluidic device for producing MEPCM fibers. ii) High‐speed images of a coaxial core–sheath flow consisting of a cylindrical jet of melting RT27 (core fluid) and an annular jet of PVB solution (sheath fluid) at various inner flow rates. Scale bar is 200 mm. Reproduced with permission.^[^ ^77^ ^]^ Copyright 2015, Elsevier. B) Schematic illustration of composite phase change microfibers with a core–sheath structure using a microfluidic fabrication process. i) Micro‐device for producing composite microfibers. ii) Cross sections of the composite microfibers illustrating the solvent extraction process. Reproduced with permission.^[^ ^78^ ^]^ Copyright 2018, Springer.

The MEPCM fibers are designed with a core–sheath structure, where the PCM and protective layer are used as the core and sheath, respectively.^[^ ^75^ ^]^ This structure allows the fibers to fully utilize the latent heat property of the PCM for temperature regulation and heat storage.^[^ ^76^ ^]^ To increase the productivity of MEPCM fibers, microfluidic technology is used in the manufacturing process. The process involves two steps. In the first step, a liquid line template is created using microfluidic methods. The sheath material is then cured through subsequent processing to produce the MEPCM fibers. For example, Wen et al.^[^ ^77^ ^]^ prepared MEPCM fibers with high PCM content based on a simple and controllable microfluidic technology, as shown in **Figure** **9**A. It was found that the poly(vinyl butyral) (PVB) polymer shell of the obtained MEPCM fibers effectively prevents the leakage of RT27 during the phase transformation process and the enthalpy of MEPCM fibers increases with the increase of PCM content. The microfibers showed a high encapsulation efficiency of up to 70%, with a maximum melting enthalpy of about 128.2 J g^−1^ and a crystallization enthalpy of about 124.0 J g^−1^. To improve the thermal conductivity of MEPCM fibers, researchers can add high thermal conductivity materials such as graphene nanosheets and metal oxides to the sheath.^[^ ^78^ ^]^ Zhang et al.^[^ ^78^ ^]^ used the microfluidic method to prepare a composite MEPCM fiber with high thermal conductivity (Figure 9B). This fiber consisted of an RT27 core and a PVB sheath mixed with Al_2_O_3_ nanoparticles. The addition of 12% Al_2_O_3_ resulted in a 47.1% decrease in melting time and a 39.5% decrease in crystallization time. **Table** **4** provides a summary of the thermal properties and morphological characteristics of various MEPCM fibers.

**Table 4:** Thermal properties and morphological characteristics of some MEPCM fibers.

Ref.	Geometry	Device	Emulsions	Phases	Curing method	Core material	Shell material	Fiber diameter	Δ*H*_m_ [J g^−1^]	*T*_m_ [°C]	Δ*H*_c_ [J g^−1^]	*T*_c_ [°C]	*W* [%]	*E* [%]
[77]	Co‐flow	Glass capillary	Double	O/O/W	Solution extraction	Paraffin wax RT27	PVB	320–350 µm	128.2	27.88	–	25.13	–	70
[79]	Co‐flow	Glass capillary	Double	O/O/W	Solvent extraction	Paraffin wax RT27	MWNT/PVB	400–450 µm	108.67	30		23	–	
[75]	Co‐flow	Glass capillary	Single	W/O	Solution extraction	Solution extraction	PVB, Al_2_O_3_ NPs composites	400	73.58	12	–	6	–	96.0
[78]	Co‐flow	Glass capillary	Double	O/O/W	Solvent extraction	Paraffin wax RT27	PVB, Al_2_O_3_ NPs composites	400 µm	117.3	26.39	–	23.14	–	64.8
[80]	Co‐flow	Glass capillary	Single	O/W	–	PEG1000	polypropylene	850–950 µm	108.4		106.7	20.4	80.3	96.7

## 5 Characterization of MEPCM Capsules/Fibers

The properties of MEPCM capsules/fibers, including their physical, chemical, and mechanical characteristics, are greatly affected by the materials used for the core and shell, as well as the method used to synthesize them. These properties are important for the practical use of MEPCMs, and it is crucial to accurately measure and describe them to ensure their effectiveness.

### 5.1 Encapsulation Rate

The encapsulation rate of MEPCM capsules/fibers is an important parameter to evaluate the thermal properties of MEPCMs. MEPCM capsules/fibers prepared by microfluidic device generally have a high encapsulation rate. Tables 3 and 4 show the comparison of the encapsulation efficiency of MEPCM capsules/fibers prepared by some current microfluidic methods.

The encapsulation rate is an important parameter for evaluating the thermal properties of MEPCM capsules/fibers. The encapsulation rate of MEPCM capsules/fibers, also known as the effective load of ME‐PCM capsule/fiber, refers to the ratio of the amount of core material covered to the total amount of core material in MEPCM capsule/fiber. In theory, accurately weighing the mass of the core material in the MEPCM capsule/fiber is difficult. Generally, the melting latent heat of the MEPCM capsules/fibers measured by differential scanning calorimeter (DSC) can be compared with the melting latent heat of the pure PCM to calculate the encapsulation efficiency, as shown in Equation (8)^[^ ^31^ ^]^

(8) $$E=\frac{\Delta H_{m,\mathrm{ME}-\mathrm{PCM}\;\mathrm{capsule}/\mathrm{fiber}}}{\Delta H_{m,\mathrm{PCM}}}\times 100\%$$

where *E* is the encapsulation efficiency of MEPCM capsules/fibers; Δ*H*_m, ME − PCM capsule/fiber_ and Δ*H*_m,PCM_ are, respectively, the melting latent heat of MEPCM capsules/fibers and core material. MEPCM capsule/fiber prepared by microfluidic device generally has a high encapsulation rate. Tables 3 and 4 show the comparison of the encapsulation efficiency of MEPCM capsules/fibers prepared by some current microfluidic methods.

### 5.2 Particle Size Distribution

The particle size distribution of MEPCM capsules/fibers will directly affect their thermal and mechanical properties. For the same MEPCM capsules with the same wall thickness, the smaller the particle size is, the shorter the heat transfer distance of the core material is, and the higher the heat transfer efficiency is, but it will affect the stored energy density. At the same time, smaller particles have higher mechanical strength. Scanning electron microscopy (SEM) and optical microscopy can be used to evaluate the particle size and morphological characteristics of microcapsules, while a laser particle size analyzer can explore the particle size distribution of microcapsules.

**Figure 10:**
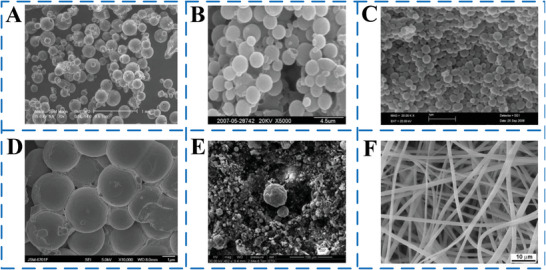
SEM micrographs of MEPCM capsules/MEPCM fiber prepared by conventional methods. A) Suspension polymerization. Reproduced with the permission.^[^ ^18^ ^]^ Copyright 2018, Springer‐Verlag. B) In situ polymerization. Reproduced with the permission.^[^ ^81^ ^]^ Copyright 2009, Elsevier. C) Emulsion polymerization.Reproduced with the permission.^[^ ^23^ ^]^ Copyright 2010, Elsevier. D) Dispersion polymerization. Reproduced with the permission.^[^ ^24^ ^]^ Copyright 2011, Elsevier. E) Interfacial polymerization. Reproduced with the permission.^[^ ^82^ ^]^ Copyright 2008, Wiley. F) Electrospinning. Reproduced with the permission.^[^ ^16^ ^]^ Copyright 2011, Elsevier.

From the SEM image shown in **Figure** **10**A–E, the MEPCM capsules prepared by conventional methods have poor monodispersity. The size of the capsules and the content of PCMs are not controllable, which will also cause a great waste of reagents and increase the preparation cost of MEPCM capsules. Figure 10F shows the SEM diagram of the MEPCM fiber prepared by the conventional electrospinning method.

**Figure 11:**
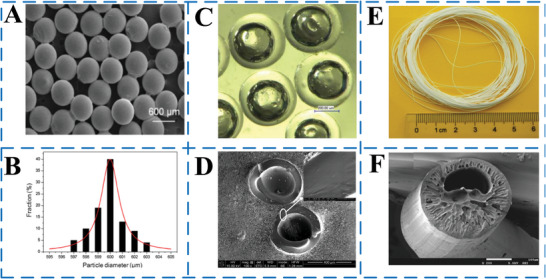
A) SEM of MEPCMs; B) particle size distribution of MEPCMs.Reproduced with permission.^[^ ^61^ ^]^ Copyright 2019, Elsevier. C) Optical microscope of MEPCMs. D) Cross‐sectional SEM image of MEPCMs.Reproduced with permission.^[^ ^59^ ^]^ Copyright 2019, Elsevier. SEM images of microfibers after the removal of paraffin RT27 fabricated at *Q*_i_ of E) 8 and F) 16 mL min^−1^. The middle (*Q*_m_) and outer flow rate are, respectively, fixed at 100 and 250 mL min^−1^. Reproduced with permission.^[^ ^77^ ^]^ Copyright 2015, Elsevier.

In **Figure** **11**A,B, an SEM image and corresponding particle size distribution are shown for MEPCMs produced using double‐emulsion droplet templating. The MEPCMs have a highly uniform size distribution, with only a 6 µm difference between the maximum and minimum sizes. The calculated coefficient of variation in size (*C*_v_) is ≈1%, indicating that combining three‐phase microfluidics and radiation curing approaches can result in MEPCMs with high uniformity and monodispersity.^[^ ^61^ ^]^ Due to the incomplete or broken outer shell of MEPCM capsules/fibers, there is a risk of leakage of internal PCMs, which would lead to the loss of protection for the core material. Therefore, it is necessary to study the morphology and microstructure of microcapsules. Due to the fine regulation of interfacial tension by microfluidics, the resulting MEPCM capsules/fibers normally have smooth surfaces and uniform morphology. The optical micrographs and SEM images presented in Figure 11C,D show that the MEPCMs created were almost uniform in size, with an average diameter of 460 mm.^[^ ^59^ ^]^ The shell of the MEPCMs was transparent and had a smooth surface, with n‐hexadecyl bromide liquid enclosed within. Although the transparency of the MEPCMs varied slightly due to uneven wall thickness, the maximum variation was only around 10 mm, as observed from the SEM images in Figure 11B. Additionally, the PVB microfibers, both with and without RT27, had uniform and smooth morphologies, as shown in Figure 11E,F. The coaxial microdevice used for fabrication allowed for the continuous production of microfibers that could reach several meters in length.

### 5.3 Thermal Conductivity

The thermal conductivity of MEPCM capsules is crucial for thermal energy storage and temperature control. A low thermal conductivity can cause a delay in the thermal response of storage and release of latent heat. Various researchers have studied the calculation method of thermal conductivity of MEPCM capsules. When MEPCM capsules are in the form of powder particles, the thermal conductivity of a single MEPCM particle can be calculated using the composite ball method,^[^ ^83^ ^]^ expressed by *k*_p_

(9) $$\frac{1}{k_{p}d_{p}}=\frac{1}{k_{c}d_{c}}+\frac{d_{p}-d_{c}}{k_{s}d_{p}d_{c}}$$

(10) $${\left(\frac{d_{p}}{d_{c}}\right)}^{3}=1+\frac{\rho_{c}\left(1-\alpha_{m,c}\right)}{\rho_{s}\alpha_{m,c}}$$

where *k*_p_, *k*_c_, and *k*_s_ are, respectively, the thermal conductivity of MEPCM particles, core material, and shell material; *d*_p_ and *d*_c_ are, respectively, the diameter of MEPCM capsules and core material; ρ_c_ and ρ_s_ are, respectively, the density of core material and shell material; α_m, c_ is the mass fraction of core material.

When MEPCM particles are dispersed in the heat exchange fluid to form MEPCM suspension (i.e., functional thermal fluid), the average thermal conductivity of MEPCM capsules suspension can be calculated using Maxwell's formula,^[^ ^83^ ^]^ which can be expressed by *k*_b_

(11) $$\frac{k_{b}}{k_{f}}=\frac{2+\frac{k_{p}}{k_{f}}+2c_{v}\left(\frac{k_{p}}{k_{f}}-1\right)}{2+\frac{k_{p}}{k_{f}}-2c_{v}\left(\frac{k_{p}}{k_{f}}-1\right)}$$

where *k*_b_ and *k*_f_ are, respectively, the thermal conductivity of MEPCM capsules suspension and carrier fluid; *c*_v_ is the volume fraction of MEPCM capsules particles in MEPCM capsules suspension.

**Figure 12:**
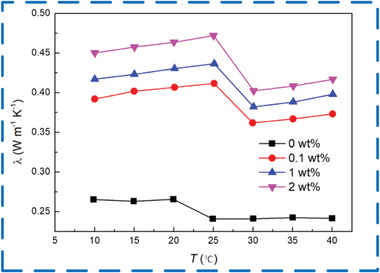
The variation of thermal conductivity of the MEPCM capsules with different mass percentage concentrations of multilayer graphene in the liquid PCM at different temperatures.Reproduced with permission.^[^ ^63^ ^]^ Copyright 2022, Elsevier.

The low thermal conductivity of traditional polymer shell materials has limited the conduction of heat energy in the MEPCM system, resulting in low thermal efficiency during heat absorption or release. This has made it difficult to store and release heat quickly and effectively, which greatly restricts the application of phase change microcapsules. To address this issue, researchers have tried various methods. One approach is to enhance the core and shell materials of MEPCM capsules. Nanomaterials such as carbon nanotubes, graphene,^[^ ^84^ ^]^ nanoalumina,^[^ ^85^ ^]^ nanosilicon nitride, and nanocopper^[^ ^86^ ^]^ can be added to the shell material to improve its thermal conductivity. In **Figure** **12**, it can be observed that the addition of multilayer graphene (≤ 2 wt%) can enhance the thermal conductivity of PCM caps while having minimal impact on the energy storage capacity (less than 5%). This allows the PCM caps to respond more quickly to changes in the surrounding thermal environment.^[^ ^63^ ^]^

### 5.4 Phase Change Heat Storage

The phase change thermal storage performance is crucial in the practical application of MEPCM capsules/fibers. It is determined by two primary parameters, namely, the phase change temperature and the phase change latent heat. These parameters can be measured using a DSC, which helps in evaluating the efficiency of the MEPCM system in storing and releasing thermal energy during phase transitions. Generally speaking, there are endothermic and exothermic peaks on the DSC temperature rise and fall curves of MEPCM capsules/fibers and phase‐change core materials, while the shell materials have no endothermic and exothermic peaks. The heat absorption and release curves of phase change core materials and microcapsules/fibers are basically unchanged in the phase change range, but the peak points of phase change appear at different positions. This is because the shell material has a certain barrier effect on the heat transfer of the core material, leading to the hysteresis of the phase change point of the core material. In addition, the average phase change latent heat of microcapsules is typically lower than the average phase change latent heat of the core material. This is because the core material is encapsulated by a shell material, which affects the heat transfer of the microcapsules/fibers and reduces the phase change latent heat. In other words, the shell material acts as a barrier that slows down the heat transfer process, resulting in a decrease in the amount of thermal energy that can be stored or released during the phase change.

**Figure 13:**
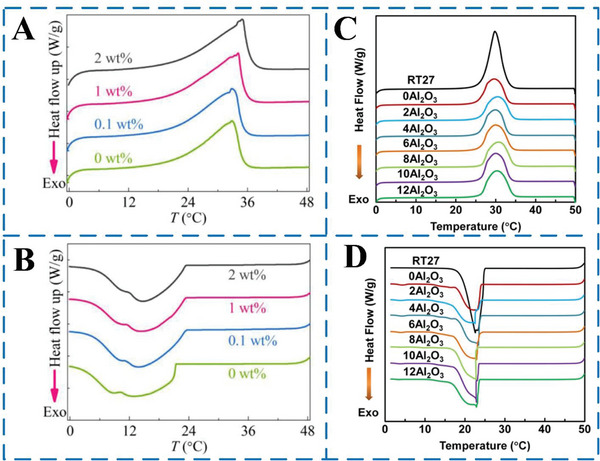
A) DSC curves of MEPCM capsules melting peaks. B) DSC curves of MEPCM capsules: solidification peaks. Reproduced with permission.^[^ ^63^ ^]^ Copyright 2022, Elsevier. C) DSC curves of paraffin RT27 and the composite MEPCM fibers in the temperature range of 0–50 °C, heating process; D) Cooling process. Reproduced with permission.^[^ ^78^ ^]^ Copyright 2018, Springer.

As mentioned earlier, scholars have attempted to add nanoparticles to microcapsules to improve thermal storage performance of MEPCM capsules/fibers. For example, Hao et al.^[^ ^63^ ^]^ conducted experiments on MEPCM capsules with varying mass ratios of multilayer graphene, subjecting them to at least two repeated phase‐change processes. The results, shown in **Figure** **13**A,B, indicate that there is a slight decrease in the proportion of PCM in the capsules as the mass ratio of multilayer graphene increases from 0 to 2 wt%. However, the loss of phase‐change enthalpy is minimal, at less than 5%, which means that the reduction in energy storage capacity is negligible. In other words, the addition of multilayer graphene does not significantly affect the energy storage performance of the MEPCM capsules. Besides, Zhang et al.^[^ ^78^ ^]^ studied the impact of Al_2_O_3_ content on the thermal energy storage and release properties of composite phase change microfibers. DSC curves were used to analyze the results, as shown in Figure 13C,D. The study found that the melting onset temperature and crystallization onset temperature of paraffin RT27 were 26.66 and 24.77 °C, respectively. When compared to RT27, the melting onset temperature and crystallization onset temperature values of the phase change microfibers with and without Al_2_O_3_ were almost identical. In other words, the addition of Al_2_O_3_ did not significantly affect the phase change temperatures of the composite microfibers. Notably, the addition of Al_2_O_3_ did not have a significant impact on the melting onset temperature and crystallization onset temperature values of the composite phase change microfibers. Additionally, the study found that the melting peak temperature and crystallization peak temperature of RT27 were 29.78 and 22.59 °C, respectively, and the addition of Al_2_O_3_ did not cause any noticeable changes in these values. In other words, the presence of Al_2_O_3_ did not significantly affect the thermal energy storage and release properties of the composite microfibers.

### 5.5 Thermal Stability

**Figure 14:**
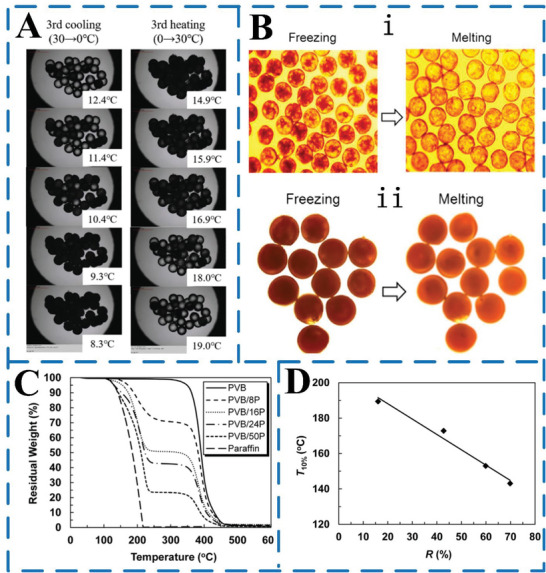
A) Optical microscope images showing the MEPCM capsules during the three‐cycle cooling–heating program. Reproduced with permission.^[^ ^62^ ^]^ Copyright 2020, Amercican Chemical Socieity. B) Typical optical microscopy images showing the surface morphologies of MEPCM capsules with the shell thicknesses of i) 30 and ii) 75 µm after 100th thermal cycling, respectively. Reproduced with permission.^[^ ^61^ ^]^ Copyright 2019, Elsevier. C) TGA curves for MEPCM fibers and paraffin RT27. D) Effect of RT27 content on T10% which is the weight of a material decreases by 10% of its original weight. Reproduced with permission.^[^ ^77^ ^]^ Copyright 2015, Elsevier.

The thermal stability of MEPCM capsules/fibers is crucial in determining their service life. If the MEPCM capsules/fibers have good thermal stability, it means they can maintain their thermal storage performance even after undergoing multiple cycles of heat release. In other words, thermal stability is an important factor in ensuring the longevity of MEPCM capsules/fibers. Currently, it can be measured by thermogravimetric analyzer (TGA). The thermal stability of MEPCM capsules/fibers is mainly affected by the microflow control preparation conditions, the type of shell structure, the type of phase change core material, and other factors. **Figure** **14**A,B shows that the MEPCM capsules prepared by microfluidic device still maintain good thermal storage performance after 100 thermal cycles of heat release.

Figure 14C,D shows the TGA curves of paraffin RT27, PVB fibers, and MEPCM fibers.^[^ ^77^ ^]^ Both paraffin RT27 and PVB fibers have only one decomposition stage, with paraffin RT27 completely decomposing at 220 °C and PVB fibers beginning to decompose at 300 °C. The phase change fibers undergo two decompositions at temperatures below 300 °C and above 300 °C, respectively. That is, the core layer RT27 decomposes at temperatures below 300 °C, and the sheath layer PVB decomposes at temperatures above 300 °C. The temperature corresponding to a 10% reduction in fiber mass (T10%) is taken as a thermal stability parameter. The T10% of two types of microfibers, RT27 and PVB, were measured and found to be 136.7 and 370.0 °C, respectively. As the ratio of the core to the sheath (*R*) increased, the T10% value of the phase change microfibers decreased linearly, approaching that of RT27. Additionally, the T10% of PVB/50P microfibers was 143.1 °C, which was 6.4 °C higher than that of RT27. These results suggest that using core–sheath‐type fibers to encapsulate paraffin RT27 can improve the thermal stability of PCMs.

## 6 The Applications of MEPCM

In recent years, MEPCM capsules and fibers have become increasingly popular in various fields, including building energy conservation, textiles, aerospace, solar energy utilization, and industrial waste heat recovery. This is due to their ability to efficiently store and release heat, as well as their flexibility in application. As a result, MEPCM materials have been widely adopted as an effective solution for thermal management in a variety of industries.

### 6.1 Buildings and Energy Savings

**Figure 15:**
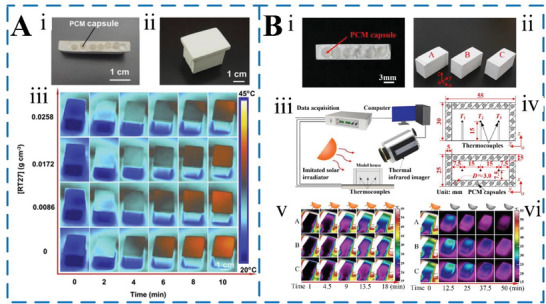
A) i) Cross‐sectional image of the gypsum board with embedded capsules. ii) A picture of a model house made of gypsum boards. iii) Thermal images of model houses made of gypsum boards containing different contents of RT27 in the roof boards. The imitated solar irradiation is 800 W m^−2^. Reproduced with permission.^[^ ^58^ ^]^ Copyright 2014, Elsevier. B) Setup for measuring the thermo‐regulation capacity of PCM capsules: i) Photograph of lossless PCM capsules inside the gypsum board after repeated melting and solidification; ii) Model houses constructed by gypsum boards with different contents (house A: pure gypsum; house B: gypsum embedded by the PCM capsules without multilayer graphene; house C: gypsum embedded by the PCM capsules with 2 wt% multilayer graphene); iii) Schematic of the IR thermal experiment; iv) Location of thermocouple inside model houses; v) IR thermal imaging of three model houses during heating process; vi) IR thermal imaging of three model houses during cooling process. Reproduced with permission.^[^ ^63^ ^]^ Copyright 2022, Elsevier.

Within a certain temperature range, PCMs can absorb, store, and release heat, regulating indoor temperature and saving energy. However, the direct application of PCMs in the phase change process is prone to leakage and corrosion, negatively impacting the strength of building materials. Wrapping PCMs in microcapsules overcomes these shortcomings. Microcapsule technology not only protects PCMs, but also makes them easy to combine with building materials, exhibiting high heat transfer efficiency and heat storage capacity. Building materials including MEPCM capsules/fibers such as cement mortar, wall, concrete, ceiling, ceramic tile, and floor hold significant potential for reducing building energy consumption.^[^ ^87^ ^]^ Liang et al.^[^ ^58^ ^]^ pioneered an efficient and adjustable technique to encase PCM within biocompatible, nontoxic calcium alginate shells. These MEPCM capsules found utility in the creation of gypsum boards. The gypsum board's cross‐sectional view displayed well‐maintained PCM capsule shapes (**Figure** **15**A‐i), indicating the robust stability of the core–shell structure within the thin capsules' walls. Model houses' roof boards, fashioned from gypsum with varying contents of PCM (RT27) but identical dimensions (Figure 15A‐ii), were subject to simulated solar radiation of 800 W m^−^^2^. Notably, Figure 15A‐iii reveals that higher [RT27] values correlated with a slower temperature rise in the roof board. This trend arises due to increased heat energy absorption during RT27's phase change process, resulting in enhanced thermal regulation. The outcomes underscored the commendable thermoregulatory potential of the developed PCM capsules. Similarly, to assess the thermoregulatory efficacy of PCM capsules, particularly those incorporating multilayer graphene, Hao et al.^[^ ^63^ ^]^ devised an experiment employing gypsum boards containing embedded PCM capsules. The gypsum board's cross‐sectional view, following repeated heating and cooling cycles, showcased the intact core–shell structures of the encapsulated PCM capsules (Figure 15B‐i). This endurance through multiple melting‐solidification cycles attested to the capsules' robust thermal stability. For a thermoregulation performance analysis, three model houses were constructed with varying gypsum board compositions (Figure 15B‐ii). House A featured pure gypsum side walls, while House B contained PCM capsules without multilayer graphene, and House C incorporated PCM capsules with 2 wt% multilayer graphene. Thermoregulation tests subjected these houses to simulated solar irradiation (500 W m^−^^2^) for PCM melting, followed by natural cooling to solidify the PCM capsules at a constant ambient temperature of 14.5 °C (Figure 15B‐ii). Results revealed that during both radiation heating and natural cooling phases, model houses B and C, equipped with PCM capsules, displayed heightened thermal inertia compared to House A without capsules. This effect was particularly pronounced during PCM melting (Figure 15B‐iii,iv) and solidification (Figure 15B‐v,vi) stages. Instead of a concrete wall, Jeong et al.^[^ ^88^ ^]^ prepared MEPCM/coating composite for thermal storage tiles to reduce the heat island effect and peak load of buildings. The research found that the size of 200 mm × 200 mm ×7 mm ceramic tile, when the mass fraction of MEPCM added is 10%, the latent heat of melting of MEPCM/hydrophobic coating is 4.826 J g^−1^, while the latent heat of melting of MEPCM/hydrophilic coating is 6.854 J g^−1^, and its thermal performance is better. Until now, the application of MEPCM in the field of building energy conservation has a good application prospect, but the realization of commercial large‐scale application still needs more research.

### 6.2 Textiles

**Figure 16:**
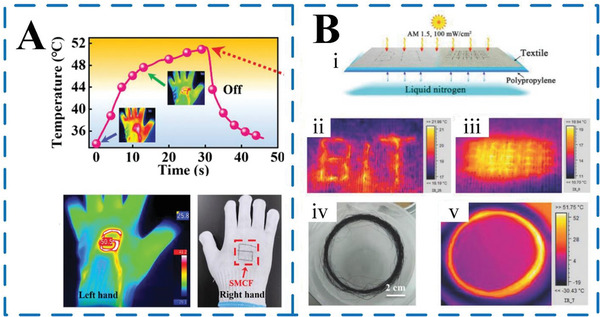
A) The thermal images of SMCF in a glove with the applied voltage of 0.4 V. Reproduced with permission.^[^ ^91^ ^]^ Copyright 2022, Wiley. B) Photograph and IR images of the ASF under solar irradiation. i) Schematic and IR images of the photonic response of ASF textile in a cold environment (selecting a temperature of ≈0 °C), ii,iii) single ASF/cotton woven fabric and iv,v) a fiber bundle placed on a cold polypropylene (PP) surface under solar illumination of 1.0 Sun. Reproduced with permission.^[^ ^92^ ^]^ Copyright 2014, Wiley.

In the 1980s, MEPCM capsules began to be used in temperature regulating textiles, achieving automatic temperature regulation by utilizing the characteristics of PCMs themselves that can store and release energy.^[^ ^89^ ^]^ It was initially used in astronaut clothing, and later gradually became civilian and diversified. Nowadays, a series of textile and clothing products have been developed, such as ski clothing, firefighting clothing, military clothing, and so on. Textiles containing phase change microcapsules have an automatic, reversible, and infinite dynamic thermal insulation function of absorbing and releasing heat, thereby forming a microclimate with a basically constant temperature around the human body. The application of MEPCM in protective textiles can effectively reduce the heat pressure and improve the comfort of clothing. In the past, conventional protective clothing used coated fabrics with poor moisture permeability and permeability, which would hinder the transmission of heat and moisture, and then lead to a significant rise in the temperature and relative humidity of the underwear microclimate, resulting in a rise in the skin temperature of the staff. The poor thermal and wet comfort experience would affect the work efficiency.^[^ ^90^ ^]^ For this reason, a recent study introduced stretchable fibers composed of a highly conductive liquid metal, achieved through a continuous large‐scale fabrication process utilizing coaxial wet‐spinning.^[^ ^91^ ^]^ These sheath–core fibers exhibit exceptional stretchability (up to 373%) and conductivity, comparable to commercial metal wires. Due to their mechanical properties and sensitivity, these fibers find diverse applications in wearable electronics. For instance, they can be seamlessly woven into gloves, forming logos that attain a temperature of 50.5 °C at 0.4 V when worn by an individual (**Figure** **16**A). Similarly, Li et al.^[^ ^92^ ^]^ successfully engineered graphene‐aerogel composite fibers with remarkable flexibility, strength, and self‐cleaning properties, offering versatile functionalities, including tunable thermal conversion and storage under various stimuli. These fibers, comprising porous graphene aerogel and organic PCMs coated with hydrophobic fluorocarbon resin, offer an expansive spectrum of phase transition temperatures and enthalpies. Investigating their photo‐to‐heat response, the study focused on low‐temperature conditions (around 0 °C) under simulated solar illumination (Figure 16B‐i). Employing a single aerogel spun fiber (ASF) fashioned into both a “BIT” letter pattern and a network fabric configuration (Figure 16B‐ii,iii), they achieved surface warming to a human‐comfortable temperature (≈19–21 °C) under solar irradiation. This was in stark contrast to the frigid ambient surroundings, signifying effective photoheat conversion and storage along the single fiber despite the cold environment. Impressively, when an ASF fiber bundle was placed directly on a cold surface, its temperature soared to 45–50 °C (Figure 16B‐iv,v), more than double the temperature increase observed in single ASF/cotton fabrics under identical solar exposure conditions.

### 6.3 Military Aviation

**Figure 17:**
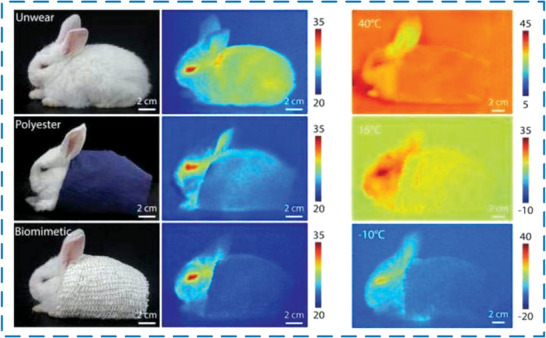
IR stealth effect of different fabrics. Reproduced with permission.^[^ ^94^ ^]^ Copyright 2022, IOP publishing Ltd.

With the continuous development of the military aviation industry, various types of military aviation equipment have increasingly high requirements for system thermal control. As early as the 1980s and 1990s, TRDC Company of the United States, with military funding, began researching phase change microcapsules for cooling systems such as aircraft and electronic components.^[^ ^93^ ^]^ In order to reduce the damage caused to electronic components by thermal mutation environments, some researchers have incorporated MEPCM into certain substrates and coated them on the surface of objects, which can maintain thermal insulation and constant temperature, improving their lifespan and reliability. Ng et al.^[^ ^4^ ^]^ successfully developed MEPCM capsules with a high thermal conductivity and electrical insulation housing. The thermal conductivity of the MEPCM capsules had been increased by 116.0%, and the resistance was higher than 1010 Ω, meeting the requirements for sufficient insulation strength. In addition, after adding MEPCM, the temperature in the center of the battery module can be reduced by 7.3 °C, which can extend its service life. Therefore, MEPCM capsules are also suitable for use in electronic devices such as batteries. At the same time, with the rapid development of IR detection technology, MEPCM also has important significance in military IR camouflage. As shown in **Figure** **17**, by adding MEPCM capsules to the fiber or coating it on the surface of the fiber, a fabric with IR stealth function was produced,^[^ ^94^ ^]^ which can effectively reduce IR thermal radiation.

### 6.4 Solar Energy Utilization

**Figure 18:**
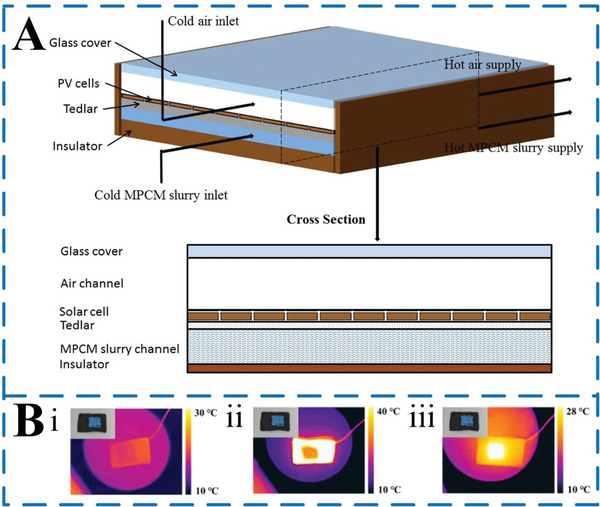
A) The schematic diagram of the integrated PV/T collector. Reproduced with permission.^[^ ^98^ ^]^ Copyright 2017, Elsevier. B) Photograph and IR images of TPCF/2 woven in a carbon fabric i) in the initial state before sunlight irradiation, ii) under sunlight irradiation, and iii) in the natural cooling process. Reproduced with permission.^[^ ^99^ ^]^ Copyright 2022, Elsevier.

PCMs have the advantages of low price, high heat storage density, and high phase change latent heat. They can significantly improve the energy storage efficiency of solar energy storage devices and reduce costs, so they can be widely used in the field of solar energy storage. Adding MEPCM capsules to building materials such as wall panels, ceilings, and bricks can absorb solar energy during the day and release it at night to maintain stability at room temperature. In addition, MEPCM capsules can be used in solar water heating systems^[^ ^95^ ^]^ and solar air heating systems.^[^ ^96^ ^]^ Using MEPCM suspension as a heat transfer and storage medium for solar collectors can enhance the absorption and utilization of solar energy.^[^ ^97^ ^]^ Besides, Liu et al.^[^ ^98^ ^]^ applied MEPCM to dual channel solar photovoltaic collectors to improve the thermal and electrical performances, as shown in **Figure** **18**A. It was found that when the concentration of MEPCM is 10%, the latent heat can reach 175 J g^−1^, and the total efficiency of photovoltaic collectors is 80.57%, which was about 1.8% higher than conventional collectors, indicating that MEPCM has the potential for further development in the field of solar collectors. Furthermore, incorporating MEPCM capsules/fibers into fabrics for solar energy harvesting represents another application beyond building materials. A notable instance is highlighted by Niu et al.,^[^ ^99^ ^]^ who employed a wet‐spinning core–sheath approach to create smart thermochromic phase change fibers (TPCFs) by directly encapsulating PCMs within flexible fibers. Under sunlight, TPCF/2 and carbon fibers initially exhibited a similar temperature of 18 °C (Figure 18B‐i). Post‐sunlight exposure (100 mW cm^−2^), carbon fibers swiftly surged to over 40 °C (Figure 18B‐ii). Upon sunlight removal, TPCF/2 rapidly cooled, stabilizing at 28 °C with evident phase change behavior (Figure 18B‐iii). Impressively, the calculated solar‐thermal energy storage efficiency (*η*_S_) reached 91.4%, surpassing a previous 82.8% efficiency in a conductive nonwoven energy storage fabric. This underscores the exceptional solar energy harvesting potential of the TPCF/2‐coupled carbon woven fabric.

### 6.5 Catalysis

**Figure 19:**
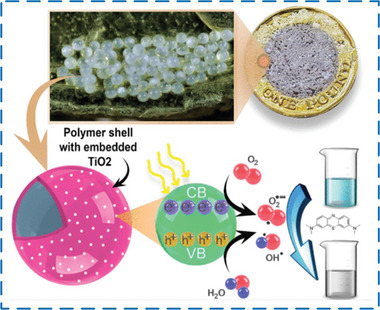
Photocatalytic activity of MEPCM capsules loaded with TiO_2_. Reproduced with permission.^[^ ^100^ ^]^ Copyright 2018, Amercian Chemical Society.

PCMs possess the unique property of being able to store thermal energy, which can be utilized to enhance the performance and stability of specific catalysts. By integrating a PCM and a catalyst into a single material, it is possible to create a self‐heating catalyst that can store thermal energy from renewable sources like solar energy or residual heat energy. This can result in an improved catalyst performance, as the stored thermal energy can be utilized to enhance the catalytic reaction. Thus, the combination of PCMs and catalysts can lead to the development of innovative materials with potential applications in various industries. In a study conducted by Nuumani et al.,^[^ ^100^ ^]^ as shown in **Figure** **19**, TiO_2_ nanoparticles were immobilized within poly(HDDA)‐based polymer microspheres using microfluidic droplet generation and on‐the‐fly photopolymerization. The fast polymerization reaction resulted in a uniform distribution of TiO_2_ within the polymer network. The resulting microspheres, which contained 0.5 wt% TiO_2_, exhibited excellent photocatalytic activity. Under UV light irradiation, they were capable of degrading 80% of methylene blue in just 9 h. Moreover, the microspheres could be easily separated from water and reused without any loss in activity. Additionally, a composite polymer shell consisting of TiO_2_ and poly(HDDA) was used to enclose an aqueous or liquid paraffin core, resulting in bifunctional microcapsules. The microcapsules exhibited high encapsulation efficiency and increased thermal stability compared to the pure polymer matrix. This research demonstrated the potential of combining PCMs and catalysts in a single material to create self‐heating catalysts with improved performance and stability. Similarly, Parvate et al.^[^ ^72^ ^]^ utilized a straightforward, reusable glass capillary microfluidic device with three phases, drawing inspiration from Lego, to create versatile microcapsules. These microcapsules, enriched with TiO_2_ NPs in their shells, demonstrated dual functionalities, simultaneously harnessing photocatalytic and thermal energy storage capabilities.

### 6.6 Biomedical Science

**Figure 20:**
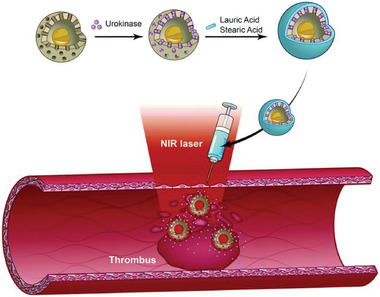
The system provides localized hyperthermia for deep vein thrombosis therapy. Reproduced with permission.^[^ ^103^ ^]^ Copyright 2020, Royal Society of Chemistry.

In recent years, there has been a growing interest in the application of organic PCMs in the field of biomedical sciences, particularly those derived from fatty acids. These organic PCMs, such as fatty acid‐based PCMs, have gained popularity due to their affordability, excellent chemical stability, and biocompatibility.^[^ ^101^ ^]^ However, a significant challenge has been the narrow range of phase transition temperatures exhibited by single‐component fatty acid PCMs, making them less suitable for use within the human body. To address this limitation, researchers have introduced the concept of organic fatty acid eutectic PCMs (EPCMs). For instance, Zhu et al.^[^ ^102^ ^]^ proposed a binary fatty acid mixture, combining lauric acid (LA) and stearic acid (SA) in a 4:1 ratio, resulting in an EPCM with a melting temperature of 39 °C. This innovative material was employed as a near‐infrared (NIR)‐triggered drug release system. When exposed to NIR radiation light, the photothermal effect raised the temperature, causing the EPCM to melt and release the drug. Similarly, Xu et al.^[^ ^103^ ^]^ designed binary eutectic phase‐change fatty acids, using LA and SA, to seal pores in gold mesoporous silica core–shell nanoparticles for the controlled delivery of thrombolytic drugs. These particles exhibited a remarkable photothermal effect triggered by NIR, facilitating rapid drug release. Furthermore, localized hyperthermia induced by the same process contributed to thrombus dissolution, offering promising prospects for clinical drug delivery, as shown in **Figure** **20**.

## 7 Perspectives and Opportunities

As is evident throughout this review, microfluidics offers precise control over the encapsulation process, resulting in uniform and well‐defined microcapsules with desired properties. Various microfluidic techniques have been developed, allowing for the encapsulation of different PCMs within protective shells. The morphology of microcapsules can be tailored, and thermal properties can be enhanced through the incorporation of additives. Nevertheless, there are still several challenges that need to be addressed to optimize the design of MEPCM capsules/fibers, which include investigating new materials for the core of the MEPCM capsules/fibers, developing new microfluidic chip, scaling up production for commercialization, and exploring new applications for these microcapsules.

- *Investigating new materials for the core of the MEPCM capsules/fibers*: The majority of MEPCM capsules/fibers produced using microfluidic control techniques are typically prepared at room temperature. This requirement poses a limitation on the choice of PCMs for the core of these MEPCM capsules/fibers, as they must be in liquid form at room temperature.^[^ ^57^, ^59^, ^61^, ^63^, ^77^, ^100^ ^]^ Consequently, the current selection of core materials is relatively limited. However, there is an exciting potential for future advancements by manipulating the working temperature during the encapsulation process. This opens up opportunities for incorporating different types of PCMs with diverse properties and applications. For instance, by adjusting the working temperature, it becomes possible to consider low melting point alloys as potential core materials, which possess advantageous characteristics, such as high thermal conductivity and excellent heat storage capacity, making them suitable for applications such as thermal energy storage and heat transfer systems. Besides, fatty acids are another class of core materials that can be explored. Fatty acids exhibit phase change behavior at relatively low temperatures, making them suitable for various applications, including thermal regulation, energy storage, and controlled release systems.
- *Developing new microfluidic chips for MEPCM capsules/fibers preparation*: At present, the microfluidics chips used for MEPCM capsules/fibers preparation are complex and expensive due to precision manufacturing techniques, specialized materials, multilayered structures, and integration of components, which contribute to increased complexity and costs.^[^ ^61^, ^63^, ^70^, ^77^, ^100^ ^]^ As a result, it is crucial to explore new chip designs that are simpler and more cost‐effective. One promising alternative is the use of modular microfluidics for the production of MEPCM capsules/fibers.^[^ ^104^ ^]^ One of the major benefits of using modular microfluidics systems is its simplicity in chip assembly.^[^ ^104^ ^]^ Unlike traditional microfluidic chip designs, which can be complex and require intricate fabrication processes, the modular microfluidic system can be easily assembled and disassembled using commercially available components. This reduces the overall cost and complexity of chip production. Moreover, the modular microfluidic system offers reusability, which can be easily disassembled and cleaned, allowing for multiple uses without the need for frequent replacement. This not only reduces the cost associated with chip materials but also minimizes waste generation.
- *Scaling up the production process from lab‐scale to commercial‐scale*: Currently, the preparation of MEPCM capsules/fibers using microfluidic control is primarily limited to laboratory‐scale production, making it difficult to generate large quantities of MEPCM capsules/fibers.^[^ ^61^, ^63^, ^78^, ^105^ ^]^ One method that shows promise for scaling up the production is by utilizing microfluidics chips with a parallel geometry. The parallel geometry of microfluidics chips allows for the simultaneous production of multiple microcapsules, increasing the production rate and efficiency. By designing the chip with parallel channels or compartments, it becomes possible to process multiple samples in parallel, thereby increasing the throughput. However, it is important to note that the parallel system of traditional microfluidics chips can be complex and expensive to manufacture. As a result, ongoing research and development should be focused on simplifying the manufacturing process and reducing costs while maintaining the benefits of the parallel geometry. By optimizing the design and fabrication techniques, it may be possible to make the production of MEPCM capsules/fibers on a larger scale more accessible and cost‐effective.
- *Developing new applications*: As discussed above, MEPCM capsules/fibers are not limited to building, textiles, solar energy utilization, and military aviation; they can also serve as multifunctional materials for both catalysis and energy storage by adding nanomaterials.^[^ ^100^ ^]^ Therefore, the exploration of multifunctional MEPCM capsules/fibers can be a promising direction. MEPCM capsules/fibers can combine the thermal energy storage capacity of PCMs with other functional characteristics of added nanomaterials. For instance, by adding nanomaterials with good electrical conductivity, such as metal nanoparticles or conductive polymers, MEPCM capsules/fibers can be endowed with conductive functions. Such multifunctional microcapsules can be used for energy storage in electronic devices, such as supercapacitors or batteries. By adding nanomaterials with sensing properties, such as magnetic nanoparticles or photosensitive nanoparticles, phase change microcapsules can respond to external stimuli and be used to prepare controllable release systems or intelligent sensors. By introducing nanomaterials with fluorescent properties, such as quantum dots or fluorescent dyes, phase change microcapsules can be used for labeling and tracking applications, such as biomedical imaging and biosensing.

## 8 Conclusions

This review summarizes the synthesis templates, microfluidic controlled preparation methods, characterization of MEPCM capsules/fibers, and their application fields. In terms of synthesis templates, the formation mode of double emulsions/liquid lines based on microfluidics was introduced. The preparation methods focused on co‐flow, flow‐focusing, and double cross‐sectional microfluidic devices. The application and development of these preparation methods were analyzed. Subsequently, the characteristics of MEPCM capsules/fibers in terms of morphology, encapsulation ratio, particle size distribution, thermal conductivity, phase change energy storage properties, and thermal stability were summarized. Finally, this paper described the application status and prospects of MEPCM capsules/fibers in the fields of building energy conservation, temperature regulating textiles, military aviation, and catalysis.

## Conflict of Interest

The authors declare no conflict of interest.
